# Association Between Hormonal Factors and Risk of Lung and Upper Aerodigestive Tract Cancer in French Women: The E3N Prospective Cohort Study

**DOI:** 10.1002/cnr2.70223

**Published:** 2025-06-09

**Authors:** Yawo E. Klu, Hélène Amazouz, Marianne Canonico, Pascal Guénel, Marina Kvaskoff, Gianluca Severi, Loredana Radoi, Aviane Auguste

**Affiliations:** ^1^ Université Paris‐Saclay, UVSQ, Inserm, Gustave Roussy, CESP Villejuif France; ^2^ Department of Epidemiology, Biostatistics and Occupational Health School of Population and Global Health, McGill University Montréal Québec Canada

**Keywords:** France, head and neck cancer, hormonal factors, lung cancer, never smokers, oesophageal cancer, oestrogen, oral contraception

## Abstract

**Background:**

Significant sex disparities exist in the incidence of lung and upper aero‐digestive tract (UADT) cancers. Inverse relationships have often been observed between exposure to hormonal factors and these cancers. Data from epidemiological studies are still inconsistent.

**Aims:**

We investigated the association between hormonal factors and the risk of cancers of the lung and UADT in French women.

**Methods:**

E3N is a French prospective population‐based cohort that recruited 98 995 women in 1990. We used a Cox proportional hazards regression models to estimate hazard ratios (HRs) and 95% confidence intervals (CIs) with age as the time scale. We also conducted a cluster analysis using the K‐means method to assess distinct patterns of hormone exposure and the cancer risk associated with them.

**Results:**

91 114 women were included (398 lung and 157 UADT). We highlighted 6 distinct exposure patterns of hormonal exposure, but no significant association was noted with cancer risk. Concerning individual factors, shorter menstrual cycles (≤ 24 days) were associated with a higher risk of developing female lung cancer even among never smokers (HR = 1.75, 95% CI = 1.01–3.01). Among never smokers, the risk of UADT was lower among women with at least 3 pregnancies compared to those with none (HR = 0.43, 95% CI = 0.20–0.91). Menarche under 12 years was associated with a non‐significant increase in the risk of UADT cancer among never smokers (HR = 1.76, 95% CI = 0.95–3.26).

**Conclusion:**

Menstrual cycle length was significantly associated with higher risk lung cancer, while UADT cancer was inversely associated with the number of pregnancies. Our findings are widely consistent with the commonly adopted hypothesis of oestrogen deficiency as a mechanism for UADT risk but not for lung cancer. While larger studies are needed to confirm these findings, our study is novel, particularly for UADT cancer since our study is one of the first longitudinal studies among never smokers.

**Trial Registration:** clinicaltrials.gov identifier: NCT03285230

**Abbreviations**
BMI: body mass index
CI: confidence interval
E3N: Etude Epidémiologique de femmes de la Mutuelle Générale de l'Education
HNC: head and neck cancer
HPV: human papillomavirus
HR: hazards ratio
IARC: International Agency for Research on Cancer
INHANCE: International Head and Neck Cancer Epidemiology Consortium
MGEN: Mutuelle Générale de l'Education Nationale
UADT: upper aerodigestive tract
VIF: variance inflation factor

## 1 Introduction

Cancers of the lung and upper aero‐digestive tract (UADT) represent a significant proportion of deaths from malignancies around the world [1, 2, 3]. The major risk factor common to these cancer sites is tobacco smoking. UADT cancers are also associated with other risk factors including alcohol. Certain risk factors such as HPV are associated with only a subset of UADT, particularly oropharyngeal cancer. Likewise, red meat and hot tea drinking are associated with an increased risk of oesophageal cancer [2, 3].

These cancers affect disproportionately men compared to women. Lung and UADT cancers in particular have some of the greatest sex disparities among all non‐reproductive cancers [4]. The male–female ratios for age‐adjusted incidence rates were 4.5 for oesophageal cancer, 2.8 for head and neck cancer and 1.5 for lung [4]. The literature has largely suggested in the past that these sex disparities were due to differences in tobacco smoking prevalence [4]. However, there is growing evidence that shows an increase in the incidence of lung and UADT cancers among never smokers and particularly among women. Moreover, women have also been described as being less sensitive to tobacco‐dependent lung and UADT cancers compared to men [1, 5]. This male predominance is largely unexplained by known risk factors. The factors driving these sex disparities are still poorly understood [6].

Epidemiological data on lung and UADT cancers suggest that attention should be focused on the disparities by sex to gain a better understanding of the disease aetiology [4]. Studies have underscored a greater role for sex steroid hormones in the carcinogenesis of lung and UADT cancers [7]. At the hormonal level, oestrogen receptor‐β (ERβ) function is required for normal morphogenesis of tissues of the lung and head and neck [8, 9].

Data from epidemiological studies thus far have shown inconsistent results [10, 11]. The most recent evidence presented in these studies is in favour of a significant inverse relationship between exposure to oestrogen‐related hormonal factors and cancers of the lung [11, 12, 13, 14, 15, 16, 17, 18, 19, 20] and head and neck [10, 21, 22, 23, 24, 25, 26, 27]. Data also support the significant role of hormonal factors in the risk reduction of lung and UADT cancers even among smokers of tobacco [10, 11]. These studies had however considerable methodological limitations in their study design and measures.

Previous studies did not assess hormone exposure in detail or the lifetime trajectory. In addition, associations were not consistently measured among never smokers across studies [11]. Furthermore, studies on UADT cancers were limited mostly to retrospective studies, which are prone to recall bias. To date, the only prospective cohort study on head and neck cancer and hormonal factors was conducted in women from the USA that showed a significant inverse relationship with hormone replacement therapy [27].

We aimed to investigate the association between hormonal factors and lung and UADT cancer risk in the E3N cohort, a prospective cohort of 100 000 French women. We assessed particularly hormonal factors linked to oestrogen exposure, namely age at menarche, menstrual cycle length, age at menopause, duration of reproductive life, number of children/pregnancies, oral contraceptive use and hormone replacement therapy.

## 2 Methods

### 2.1 The E3N Cohort

E3N is a large ongoing French prospective cohort of women set up in France in 1990 (clinicaltrials.gov identifier: NCT03285230). It comprises 98 995 women born between 1925 and 1950 from the French national health insurance plan for the national education system, the Mutuelle Générale de l'Education Nationale (MGEN). Women were enrolled in the cohort through a self‐administered questionnaire and were followed every 2–3 years on health and lifestyle. To date, over ten self‐administered questionnaires have been sent and follow up is still ongoing. Participation included providing permission to obtain information about each participant's vital status, address changes and medical expense reimbursements from the insurance plan. The average response rate to a follow‐up questionnaire was 83%, with a total loss to follow‐up of < 3% since 1990. The detailed protocol has been described elsewhere [28]. The E3N cohort was approved by the French National Commission for Data Protection and Privacy (Commission Nationale de l'Informatique et des Libertés: n°327 346); all participants gave written informed consent. The study was performed in accordance with the Declaration of Helsinki.

### 2.2 Study Population

Of the 98 995 participants enrolled in the E3N study from its inception (1990), 7781 women were excluded from the present analyses because of lost to follow‐up from baseline inclusion (Questionnaire 1: Q1). Each subsequent follow‐up period is denoted by a questionnaire number (i.e., Questionnaire 2, Questionnaire 3 etc.: Q2, Q3). Therefore, our analyses were based on 91 114 women, 554 of whom developed a respiratory cancer (lung: 398 cases and UADT: 157 cases) at Q11 (2014). The mean follow‐up of participants was 24 years. One woman was diagnosed with two separate primary tumours of the lung and UADT. The UADT cancer subsites included in our study were cancers of the oral cavity (*n* = 43), pharynx (*n* = 35), larynx (*n* = 21), salivary glands (*n* = 23), sinonasal cavities (*n* = 6) and oesophagus (*n* = 31).

### 2.3 Validation of Cancer Cases

Cancer cases were identified mainly through self‐administered questionnaires. E3N participants were asked for their physicians' addresses and permission to contact them. Self‐reported cancers were validated based on data from pathology reports or medical records. Cancers were also identified from next‐of‐kin spontaneous reports or through information from the national death registry.

### 2.4 Endogenous Hormonal Factors

Age at menarche, duration of menstrual cycle and its regularity were collected at inclusion and for subsequent questionnaires up to questionnaire 7 (Q7‐2002). Parity, number of pregnancies (including births, miscarriages, ectopic pregnancies and induced abortions) were collected in the 1990 and 1992 questionnaires. Menopausal status and age at menopause were available for inclusion and questionnaires Q4‐1995 to Q8‐2005. Study participants were asked to fill out one of four categories for their menstrual cycle length. The cutoffs were based on female menstrual physiology. The category, ‘25–31 days’ (reference) represents the range of values that are closest to the average days for a menstrual cycle in females. ‘< 24 days’ and ‘≥ 32 days’ represent shorter and longer cycles relative to the average number of days. Irregular cycles represent cycles that consistently vary in length from month to month.

### 2.5 Exogenous Hormonal Factors

Information on the use of oral contraceptives (OC) was collected at the beginning of the study. These same variables were collected for the use of hormonal treatment of menopause. These data were available from the initial inclusion until 2008.

### 2.6 Covariates

The education level of women was collected in the first questionnaire. This variable was classified into 3 categories in our study: (1) primary (no formal schooling or *certificate d'études*), (2) secondary (*Brevet du college*, technical/vocational training) and (3) tertiary education (French Baccalaureate or higher learning degrees).

Information on the smoking status (never/ever) was collected at each questionnaire. Information on alcohol consumption (g/day) was collected in the questionnaires: Q3‐1993, Q5‐1997, Q8‐2005. This variable was classified into 3 categories in our study: (‘< 5 g/day’, ‘5–25 g/day’ and ‘> 25 g/day’).

Dietary data were collected at Q3‐1993 and Q8‐2005 using a previously validated semi‐quantitative food frequency questionnaire including 208 food items including alcohol [29]. We used this questionnaire to build scores of adherence to the Mediterranean diet and Western diet [30]. Participants were divided into two groups based median score of the study sample.

The town of work was collected and defined in rural and urban areas according to the classification of the ‘Institut national de la statistique et des études économiques (INSEE)’. Hence, towns with more than 10 000 inhabitants were considered ‘urban’ and those with less than 10 000 inhabitants were considered ‘rural’. Town of work is considered as a proxy for pollution and environmental factors in this study [31].

Obesity was determined based on BMI calculation using self‐reported height and weight from the baseline. BMI was determined as weight (kg) divided by squared height (m^2^). In E3N, self‐reported anthropometry has proven reliable in a validation study [32]. Participants were split into two categories: obese (BMI ≥ 30) and non‐obese (BMI < 30).

Information on physical activity was collected on the questionnaires: Q1‐1990, Q3‐1993, Q5‐1997, Q7‐2002, Q8‐2005 and Q11‐2014. This questionnaire included questions on various activities. Each activity was assigned metabolic equivalents (METs) based on values from the Compendium of Physical Activities [33]. This score is divided into 3 categories ‘inactive’, ‘moderate’ and ‘vigorous physical activity’ (MET scores: ‘< 8.3’, ‘8.3 to 16.7’ and ‘> 16.7’, respectively) [34].

A table summarising timing of implementation of questionnaires has been described in detail elsewhere [28]. The timing used to define individual variables in the current analysis are summarised in the Supporting Information.

### 2.7 Statistical Analysis

The variables in our analyses are from measures taken at baseline (Q1) and Q3 for diet and alcohol. Our main analyses were conducted on the overall sample. We also restricted analyses to never smokers to mitigate the known confounding effect from tobacco smoking on lung and UADT cancer incidence [10]. Analyses were performed separately for lung and UADT cancer.

We performed descriptive analyses using clustering to identify distinct exposure patterns among study participants based on their lifetime exposures to hormonal factors as opposed to classical methods that examine individual exposures. To do this, we conducted a cluster analysis using the K‐means method [35] using the *tidyverse*, *cluster* and *factoextra* package available on R software (Version, 4.3.1). To carry out this analysis, we first proceeded to exclude from our analysis, women with missing data on hormonal factors selected for our study. Since this database was ultimately made up of only complete data, we proceeded to standardise the data. At the end of this process, we formed different clusters. We used the Calinski‐Harabasz index as a guiding principle to select the number of clusters that would be the most adapted to our study population [35]. We described the distribution of hormonal factor variables across clusters.

We used Cox proportional hazards regression models with age as the timescale to estimate hazard ratios (HRs) and 95% confidence intervals (CIs) [36]. We further adjusted the model for age at baseline in the regression model. The age at baseline was considered the start of the analysis, and age at cancer diagnosis (for cases) and age at last follow‐up were considered as the end point. In our analyses, we used reference categories that facilitate interpretation and that are consistent with prior epidemiological studies for better comparability (e.g., zero pregnancies).

We used two approaches to assess the exposure to hormonal factors. First, we introduced a categorical variable that divided individuals into one of six groups based on the distinct hormonal exposure patterns identified from the clustering analysis. Second, we introduced each individual hormonal exposure simultaneously as separate covariates in the same model.

The essential hormonal factors were introduced into the model after ruling out the possibility of multi‐collinearity. Multi‐collinearity was estimated by the variance inflation factor (VIF) [37]. The multivariate model was also adjusted for education level, obesity, Western and Mediterranean diet, alcohol drinking, physical activity and town of work.

To attempt to address missing data, we introduced missing categories in the regression models for variables with a considerable percentage of missing data. We acknowledge that the proportions of the missing category were large for diet and town of work (22% and 28%). However, these missing data patterns were not skewed towards the status for hormone exposure or cancer incidence. Statistical analyses were performed using the Statistical Analysis System software, version 9.4 (SAS Institute).

As a part of our sensitivity analysis, we performed a complete case analysis on the overall sample and among never smokers. We also re‐run our statistical models with the outcome variable defined as ‘being diagnosed with either lung or UADT’.

## 3 Results

### 3.1 Patient Characteristics

Table 1 shows the distribution of the hormonal factors as well as patient characteristics. The mean age at baseline was 49 years (Standard deviation = 6.6) with extremes ranging from 39 to 66 years. Ever smokers accounted for 46% of the study participants. Half of the women had their menarche between the ages of 12 and 13. Sixty‐nine percent of women had a regular cycle of 25–31 days. Menopause occurred mostly between 46 and 55 years (83.8%). The average reproductive lifespan was 38 years. Most women had 2 children (42.2%). Sixty percent of women reported ever taking oral contraceptive pills. Similarly, 64% of women were ever users of hormonal replacement therapy (HRT) for menopause. Except for oral contraceptive pills (53%), the same proportions of hormonal factors were widely observed between the overall sample and never smokers.

**TABLE 1:** Socio‐demographic characteristics and hormonal factors of participants in the study sample.

Characteristics	Overall		Never smokers	
	*n* = 91 114	%	*n* = 48 674	%
Age at inclusion				
< 49	43 207	(47.42)	20 616	(42.36)
≥ 49	47 907	(52.58)	28 058	(57.64)
Town of work				
Rural	36 052	(54.65)	20 977	(59.18)
Urban	29 921	(45.35)	14 471	(40.82)
Missing	25 141		13 226	
Education level				
Primary	4369	(4.98)	3198	(6.82)
Secondary	7729	(8.81)	4837	(10.32)
Tertiary	75 670	(86.22)	38 845	(82.86)
Missing	3346		1794	
Western diet				
Q1‐Q2	35 412	(49.80)	19 823	(52.30)
Q3‐Q4	35 702	(50.20)	18 082	(47.70)
Missing	20 000		10 769	
Mediterranean diet				
Q1‐Q2	35 585	(50.04)	19 474	(51.38)
Q3‐Q4	35 529	(49.96)	18 431	(48.62)
Missing	20 000		10 769	
Tobacco smoking				
Never	48 674	(53.44)	NA	NA
Ever	42 403	(46.56)	NA	NA
Missing	37			
Alcohol drinking (g/day)				
< 5	50 510	(55.44)	29 756	(61.13)
5–24	30 904	(33.92)	15 315	(31.46)
≥ 25	9700	(10.65)	3603	(7.40)
Missing	0		0	
BMI				
≥ 25	72 640	(82.18)	38 496	(81.56)
< 25	15 755	(17.82)	8704	(18.44)
Missing	2719		1474	
Physical activity				
Inactive	2037	(2.27)	1135	(2.36)
Moderate	7471	(8.31)	3878	(8.07)
Vigorous	80 383	(89.42)	43 015	(89.56)
Missing	1223		646	
Age at menarche (years)				
12–13	44 869	(50.45)	24 024	(50.50)
< 12	18 612	(20.93)	9566	(20.11)
≥ 14	25 464	(28.63)	13 981	(29.39)
Missing	2169		1103	
Menstrual cycle length (days)				
≤ 24	5131	(5.86)	2786	(5.97)
25–31	60 458	(69.09)	32 566	(69.76)
≥ 32	8777	(10.03)	4540	(9.72)
Irregular	13 145	(15.02)	6794	(14.55)
Missing	3603		1988	
Age at menopause (years)				
< 40	1809	(2.03)	994	(2.09)
41–45	5154	(5.79)	2784	(5.84)
46–55	74 610	(83.84)	39 853	(83.61)
≥ 55	7417	(8.33)	4035	(8.47)
Missing	2124		1008	
Duration of reproductive life (years)				
< 32	5470	(6.29)	2979	(6.39)
32–35	16 146	(18.58)	8489	(18.22)
36–38	29 712	(34.19)	15 797	(33.90)
39–41	25 164	(28.95)	13 620	(29.23)
≥ 42	10 418	(11.99)	5717	(12.27)
Missing	4204		2072	
Number of children				
0	10 850	(11.91)	5270	(10.83)
1	14 451	(15.86)	7343	(15.09)
2	38 514	(42.28)	20 586	(42.30)
≥ 3	27 288	(29.95)	15 469	(31.78)
Missing	11		6	
Number of pregnancies				
0	8439	(9.26)	4361	(8.96)
1	11 361	(12.47)	5936	(12.20)
2	28 860	(31.68)	15 685	(32.23)
≥ 3	42 443	(46.59)	22 686	(46.61)
Missing	11		6	
Oral contraceptive				
Never	36 280	(39.82)	22 706	(46.65)
Ever	54 834	(60.18)	25 968	(53.35)
Hormone replacement therapy				
Never	32 357	(35.51)	18 151	(37.29)
Ever	58 755	(64.49)	30 521	(62.71)
Missing	2		2	

*Note:* E3N cohort study, France 1990–2014.

Abbreviation: NA, Not applicable.

### 3.2 Cluster Analyses

Our cluster analysis revealed 9 distinct profiles based on the distribution of hormonal factors in the cohort. The Calinski‐Harabasz indices determined that six clusters would be the most parsimonious based on the distribution of our data. The principle of parsimony in statistical modelling emphasises favouring simpler models with fewer parameters over more complex ones, as long as both provide a comparable fit to the data. Table 2 shows the distribution of hormonal factors by cluster groups. For the sake of clarity, names were assigned based on the most atypical characteristics, but it is important not to forget the representation of other variables in the clusters. It should be noted that the group referred to as ‘reference’ includes the second largest number of individuals and appears, according to medical experts, to reflect the standard group. Cluster group 2 (reference) had a median age at menarche and menopause of 13 years and 52 years respectively. Group 2 was also characterised by an absence of irregular menstrual cycles and ever HRT use. Group 1 was the largest group in size (*n* = 27 556) with a slightly lower median age at menopause (51 years, IQR = 49–52) compared to the reference group (52 years, IQR = 50–54). All women in Group 3 experienced irregular menstrual cycle and over half of them used HRT in their lifetime. Group 4 was characterised mainly by lower parity (median = 1 child) compared to the other groups. Group 4 also had a fair amount of HRT users (68%) but a very small proportion of irregular menstrual cycle lengths (1.5%). Group 5 had a lower median age at menarche and all women in this group were ever users of HRT. Group 6 was the smallest group (*n* = 4923) and was characterised by having considerably earlier menopause (41 years, IQR = 38–44). A third of these women were ever HRT users and there were a small proportion of women with irregular menstrual cycles (10.9%). Oral contraceptive use was slightly higher (68%) in groups 1 (HRT), group 3 (irregular menstrual cycles) and group 5 (early menarche and HRT) compared to women from the other groups (range = 52%–56%).

**TABLE 2:** Summary statistics of hormonal factors for participants in the six groups identified by clustering analysis.

Hormonal factors	Group 1	Group 2	Group 3	Group 4	Group 5	Group 6
	HRT	Reference	Irregular menstrual cycles	Low parity	Early menarche and HRT	Early menopause
	*n* = 27 556	*n* = 16 808	*n* = 11 060	*n* = 10 030	*n* = 7827	*n* = 4923
Median [IQR]						
Duration of reproductive life	37.7 [35.8–39.4]	39 [37.0–41.4]	38 [36.0–40.3]	38 [36.0–40.0]	39.5 [37.5–41.1]	29 [25.5–31.5]
Age at menarche	13 [12.5–14]	13 [12–14]	13 [12–14]	13 [12–13.5]	11 [11–11]	12.5 [12–13.5]
Number of children	2 [2–3]	2 [2–3]	2 [2–3]	1 [1–1]	2 [2–3]	2 [2–3]
Number of pregnancies	3 [2–4]	3 [2–4]	3 [2–4]	1 [1–1]	3 [2–4]	2 [2–3]
Age at menopause	51 [49.0–52.6]	52 [50–54]	51 [49.58–53.1]	51 [49–52.7]	50.5 [48.5–52.0]	41.8 [38.0–44.0]
*n* (%)						
Irregular menstrual cycle	0 (0.0)	0 (0.0)	11 065 (100.0)	147 (1.5)	0 (0.0)	536 (10.9)
HRT	27 560 (100)	0 (0.0)	7246 (65.5)	6883 (68.6)	7827 (100.0)	1782 (36.2)
Oral contraceptive	18 921 (68.7)	9085 (54.0)	7539 (68.1)	5626 (56.1)	5338 (68.2)	2593 (52.7)

*Note:* Analyses performed on a subgroup of participants without missing data for variables on hormonal exposure (*n* = 78 229 women). E3N cohort study, France 1990–2014.

Abbreviation: HRT, hormone replacement therapy.

### 3.3 Association Between Hormonal Factors and Cancer Risk

We observed several statistically significant associations among the overall sample (inclusive of smokers). Age at menopause (41–45 years), shorter and irregular menstrual cycle length were associated with an increased risk of lung cancer. Whereas, later age at menarche (≥ 14 years) was associated with a reduced risk of lung cancer. We observed a non‐significant increase in lung cancer risk among ever users of oral contraceptive pills. Higher number of pregnancies was associated with a lower risk of UADT cancer. Inversely, UADT risk was increased among women who experienced menarche from 14 years onward and women who had menopause between 41 and 45 years (Table 3).

**TABLE 3:** Multivariate HR and 95% CI of lung and UADT cancer associated with hormonal factors.

	Overall						Never smokers					
	Lung (*n* = 398)			UADT (*n* = 157)			Lung (*n* = 152)			UADT (*n* = 67)		
	*n* (row%)	HR	95% CI	*n* (row%)	HR	95% CI	*n* (row%)	HR	95% CI	*n* (row%)	HR	95% CI
Endogenous factors												
Age at menarche (years)												
< 12	76 (0.41)	0.85	(0.66–1.11)	27 (0.15)	0.98	(0.63–1.54)	29 (0.30)	0.92	(0.60–1.41)	17 (0.18)	1.76	(0.95–3.26)
12–13	221 (0.49)	1	ref	65 (0.14)	1	ref	82 (0.34)	1	ref	25 (0.10)	1	ref
≥ 14	98 (0.38)	0.73	(0.57–0.92)	59 (0.23)	1.58	(1.10–2.25)	40 (0.29)	0.80	(0.55–1.17)	24 (0.17)	1.57	(0.89–2.77)
Number of pregnancies												
0	29 (0.34)	1	ref	23 (0.27)	1	ref	13 (0.30)	1	ref	10 (0.23)	1	ref
1	54 (0.48)	1.52	(0.96–2.42)	20 (0.18)	0.67	(0.37–1.24)	16 (0.27)	0.97	(0.46–2.07)	9 (0.15)	0.68	(0.27–1.69)
2	120 (0.42)	1.38	(0.91–2.11)	37 (0.13)	0.48	(0.28–0.82)	54 (0.34)	1.24	(0.65–2.33)	22 (0.14)	0.61	(0.28–1.30)
≥ 3	195 (0.46)	1.37	(0.91–2.06)	77 (0.18)	0.61	(0.37–0.99)	69 (0.30)	1.00	(0.53–1.86)	26 (0.11)	0.43	(0.20–0.91)
Age at menopause (years)												
< 40	8 (0.44)	0.96	(0.47–1.94)	1 (0.06)	0.30	(0.04–2.17)	3 (0.30)	0.93	(0.29–2.95)	0 (0.00)	NA	NA
41–45	42 (0.81)	1.85	(1.34–2.56)	17 (0.33)	1.73	(1.01–2.97)	12 (0.43)	1.35	(0.75–2.46)	6 (0.22)	1.52	(0.65–3.56)
46–55	313 (0.42)	0.68	(0.43–1.07)	125 (0.17)	0.82	(0.43–1.57)	119 (0.30)	1	ref	53 (0.13)	1	ref
≥ 55	21 (0.28)	1	ref	10 (0.13)	1	ref	13 (0.32)	1.09	(0.60–1.99)	7 (0.17)	1.34	(0.6–2.99)
Menstrual cycle length												
≤ 24 days	33 (0.64)	1.55	(1.08–2.24)	11 (0.21)	1.14	(0.60–2.19)	15 (0.54)	1.75	(1.01–3.01)	5 (0.18)	1.39	(0.55–3.52)
25–31 days	248 (0.41)	1	ref	106 (0.18)	1	ref	100 (0.31)	1	ref	42 (0.13)	1	ref
≥ 32 days	34 (0.39)	1.10	(0.76–1.57)	12 (0.14)	0.86	(0.47–1.57)	11 (0.24)	0.90	(0.48–1.68)	7 (0.15)	1.38	(0.61–3.09)
Irregular	68 (0.52)	1.32	(1.00–1.73)	20 (0.15)	0.88	(0.54–1.42)	22 (0.32)	1.10	(0.69–1.75)	9 (0.13)	1.09	(0.53–2.26)
Exogenous factors												
HRT												
Never	141 (0.44)	1	ref	61 (0.19)	1	ref	53 (0.29)	1	ref	29 (0.16)	1	ref
Ever	257 (0.44)	0.93	(0.75–1.17)	96 (0.16)	0.87	(0.61–1.24)	99 (0.32)	1.12	(0.78–1.61)	38 (0.12)	0.81	(0.48–1.37)
Oral contraceptive												
Never	165 (0.45)	1	ref	69 (0.19)	1	ref	74 (0.33)	1	ref	37 (0.16)	1	ref
Ever	233 (0.42)	1.24	(0.99–1.56)	88 (0.16)	1.06	(0.74–1.53)	78 (0.30)	1.35	(0.95–1.93)	30 (0.12)	1.04	(0.60–1.8)

*Note:* Model adjusted for all variables listed in the table, plus age at study inclusion, town of work, education level, western diet, mediterranean diet, tobacco smoking, alcohol drinking, body mass index and physical activity. Analyses performed using missing category for certain variables (*n* = 91 075 women). row%: Cumulative incidence of cancer among study participants with a given category of exposure. E3N cohort study, France 1990–2014.

Abbreviation: HRT, hormone replacement therapy.

We saw a few noteworthy changes in the statistical associations found when we restricted our analysis to never smokers. Having a shorter menstrual cycle (≤ 24 days) was associated with a significant increase of 75% in lung cancer risk (HR = 1.75, 95% CI = 1.01–3.01, *p* = 0.04) compared to women with a normal cycle (25–31 days). Despite restricting to never smokers, we still observed a non‐significant but noteworthy increase in lung cancer risk among ever users of oral contraception (HR = 1.35, 95% CI = 0.95–1.93, *p* = 0.10). Similarly, the decrease in the risk of UADT remained associated with the number of pregnancies but was only statistically significant for women who had at least 3 pregnancies. Among these women, we noted a considerable 60% reduction in UADT cancer risk (HR = 0.43, 95% CI = 0.20–0.91) compared to nulligravidae. Among never smokers, the previous association with later menarche was attenuated. Additionally, we observed a non‐significant increase in risk of developing UADT cancer among women experiencing menarche before 12 years (HR = 1.76, 95% CI = 0.95–3.26). The results obtained for univariate analyses (Table S1) and covariates in the multivariate model can be found in the Tables S2 and S3.

We were also interested in whether the distinct profiles identified from our clustering analysis were associated with respiratory cancer risk (Table 4). No statistically significant associations were observed with either lung or UADT cancer risk. However, we noted that the HR were highest for group 5 (early menarche and HRT) among both cancer types except for UADT never smokers.

**TABLE 4:** Multivariate HR of Lung and UADT cancer and 95% CI associated with cluster group.

Clusters		Lung			UADT		
	*n*	*n*	HR	95% CI	*n*	HR	95% CI
Overall							
Group 1	27 556	114	1.21	(0.74–1.98)	42	1.16	(0.59–2.27)
Group 2	16 808	70	1	ref	27	1	ref
Group 3	11 060	60	1.14	(0.61–2.13)	17	1.00	(0.41–2.42)
Group 4	10 030	48	1.21	(0.65–2.23)	16	1.80	(0.84–3.85)
Group 5	7827	34	1.66	(0.89–3.07)	13	0.97	(0.37–2.57)
Group 6	4923	30	1.55	(0.70–3.45)	10	0.72	(0.16–3.21)
Never smokers^a^							
Group 1	12 892	67	1.09	(0.53–2.25)	28	0.81	(0.32–2.06)
Group 2	7220	36	1	ref	12	1	ref
Group 3	5281	39	1.08	(0.42–2.76)	10	0.66	(0.18–2.51)
Group 4	4862	35	1.23	(0.50–3.03)	10	1.14	(0.37–3.52)
Group 5	3787	22	1.28	(0.47–3.46)	4	1.45	(0.46–4.50)
Group 6	2249	21	0.77	(0.17–3.45)	7	1.11	(0.23–5.22)

*Note:* Model adjusted for age at study inclusion, town of work, education level, western diet, mediterranean diet, tobacco smoking, alcohol drinking, body mass index and physical activity. Analyses performed on a subgroup of participants without missing data for variables on hormonal exposure (*n* = 78 229). E3N cohort study, France 1990–2014.

^a^ 25 participants with missing data for smoking status.

### 3.4 Sensitivity Analysis

We performed complete case analyses on the same multivariate statistical models. The results were largely similar to those from our main analysis where we introduced a missing category for certain variables in the model. Although the HRs were less precise, their orders of magnitude were similar, and we observed the same directions for the associations (Tables S3 and S4).

## 4 Discussion

We studied the influence of hormonal factors on the incidence of lung and UADT cancer among women from the French E3N cohort. Among never smokers, we showed that women with a menstrual cycle of ≤ 24 days were at a greater risk of developing lung cancer compared to those with cycles lasting 25–31 days. While having at least 3 pregnancies significantly reduced UADT cancer risk compared to nulligravidae. Our clustering analysis revealed six distinct profiles of hormonal exposure among our women, but no associations were observed with cancer risk.

Currently there is no consensus on the role of the menstrual cycle on lung cancer risk. Most studies so far conclude on an absence of an association with lung cancer and menstrual cycle length [15, 38, 39, 40]. Our study is one of the few to reveal a significant association between menstrual cycle length and lung cancer [41]. Our results are consistent with a meta‐analysis that showed a decrease in lung cancer risk among women with longer menstrual cycles (> 30 days) compared to those with shorter cycles (< 27 to ≤ 30 days) [41]. In terms of biological mechanisms, shorter menstrual cycle length may increase the cumulative endogenous oestrogen exposure since women with shorter cycles experience a greater number of cycles during their reproductive years [42, 43]. While these studies are in favour of a protective effect of shorter menstrual cycles, other reports suggest that shorter menstrual cycles may consistently result in higher oestrogen exposure [43, 44]. They showed that both shorter and longer cycles were associated with more anovulation relative to normal cycles. Thus, the association between shorter menstrual cycles and increased lung cancer risk is still plausible via more complex mechanisms. However, the interplay between cycle length, hormonal fluctuations and carcinogenesis is intricate, indicating the need for further research to fully understand these associations [43, 44].

Although non statistically significant, we found a 35% greater risk of lung cancer (HR = 1.35 95% CI = 0.95; 1.93) among ever users of oral contraception; contrarily, to the expected protective effect based on suspected biological mechanisms [9]. However, previous studies have found results that were consistent with the positive association that we found between oral contraception and lung cancer [45, 46]. Moreover, these data for the use of oral contraception corroborate with the increase in female lung cancer mortality among never smokers [47]. Unlike other studies [48, 49], lung cancer risk was not associated with the other hormonal factors that were examined in our study. Moreover, our findings are not consistent with the concept of oestrogen deficiency as a risk for lung cancer [9].

Unlike our results for lung cancer, those for the risk of UADT cancer are more consistent with the concept of oestrogen deficiency [50, 51]. Compared to women who were never pregnant, we found a significantly reduced risk of UADT cancer among women who had at least three pregnancies. These findings on pregnancies corroborate with data from a pooled analysis from the INHANCE consortium showing a significant reduction in head and neck cancer risk among parous women. However, a cohort study from the USA did not reveal any association with parity [27]. The biological mechanisms behind sex hormones in UADT cancer risk remain a topic of debate, though evidence suggests oestrogen may have protective effects. The plausibility of our association with the number of pregnancies is supported by a recent review on the biological mechanisms of oestrogen in head and neck cancer that highlighted an increased expression of oestrogen receptors (ERs) including nuclear ERs (ERα and ERβ) and membrane‐bound forms (ERα36, GPER1 and NaV1.2) in various head and neck squamous cell carcinomas (HNSCC) [52]. Furthermore, Oestradiol (E2), the predominant oestrogen during the reproductive period, has been shown to attenuate the growth and viability of HPV‐positive cancers in vitro, though not HPV‐negative ones [53]. Bristol et al. identified two mechanisms by which E2 suppresses tumour growth in HPV‐positive cancers: (1) repression of the viral long coding region and (2) sensitization of cells through E6 and E7 HPV gene expression [53].

We also found a significant association with age at menarche in the overall sample. A pooled analysis from the INHANCE consortium showed that menarche at 14 years and over was associated with a greater risk of head and neck cancer compared to less than 14 years [10]. These data are consistent with our findings in the overall sample but not among never smokers. Although non‐significant, our analysis among never smokers revealed an increased risk of UADT cancer in women who experienced menarche at earlier ages (< 12 years). Few studies have been able to study age at menarche as multiple distinct categories. Therefore, this positive association for earlier menarche is novel and raises questions of a potential non‐linear relationship with age at menarche and UADT cancer where the risk is greater among women who experience menarche outside the 12–13 years range.

In terms of exogenous hormonal exposure and UADT cancer risk, we did not observe any significant association. A cohort study from the USA and a systematic review both reported an inverse association with head and neck cancer and HRT use [25, 27]. Whereas HRT and oral contraception use appeared to increase the risk of oral cancer in a Korean study [54]. The discordance between previous studies and our results may be due to the inconsistency in the anatomical sites and the histological types included across studies. We grouped head and neck cancers with oesophageal cancer regardless of histological type whereas the previous above‐mentioned studies focused their work on squamous cell carcinomas of either head and neck only [25] or including the oesophagus [27].

Tobacco smoking is an important factor to consider for respiratory cancer aetiology because of its known confounding effect. Relatively few studies focused on restricting samples to never smokers, so this could hinder comparability with previous studies [38]. We showed that the role of tobacco smoking on the effect of hormonal factors is noteworthy in our study. When we restricted analyses to only never smokers, we saw that the association dissipated for menstruation (lung cancer) and age at menopause (both lung and UADT).

Clustering analyses were performed to examine whether there were certain distinct profiles of hormonal exposure that would be more at risk for cancer rather than isolating specific exposures. None of the distinct profiles of hormonal factors identified through the K‐means methods were associated with cancer risk in our study. Given the number of regression parameters to be estimated and the low number of cancer cases, it is probable that statistical power could have been insufficient to show significant differences for these clusters. Also, the exclusion of subjects with missing data on selected hormonal variables in the cluster analyses may have introduced bias, particularly in cases where missing data were not randomly distributed. However, in our study, missing data patterns were not associated with cancer incidence and therefore, selection bias is less likely. Nevertheless, we believe that these findings from our clustering analysis are a valuable addition to the knowledge base on this topic. To our knowledge, this is the first time that this clustering approach has been used to estimate the effect of hormonal factors on lung and UADT cancers [10, 27, 38]. Given the discordance between studies on examining factors individually, greater consideration could be given to looking at hormonal factors (endogenous and exogenous) in a more holistic manner as we did in this current study.

We acknowledge our study limitations. E3N is indeed one of the largest and longest prospective cohorts in France. However, this is a cohort of women in the French public education service. Moreover, women of a higher socioeconomic class are overrepresented in this sample compared to the general French population. Therefore, our findings may not be generalizable to the wider female population. Selection bias is also probable because of the missing data on the hormonal factors and adjustment variables. Also, our study did not take into account the different types of OC, nor hormone replacement therapy of menopause, which have previously been reported as determinants of cancer risk [55]. We cannot exclude the possibility of information bias for menstrual cycle length. However, more than half of the study participants were still menstruating at the time, and therefore the recall was limited to a recent event. We acknowledge that our analytic approach may obscure potential differences between menopause types (natural and artificial menopause including surgical). Therefore, it is harder to draw conclusions from our results on the number of pregnancies, age at menopause and hormone use. We did not differentiate between menopause types in our analysis, as women with artificial menopause (including surgical causes) represent only approximately 7% of the study population. Given the limited number of cancer cases overall, analyses by menopause type yielded unstable estimates (data not shown). We therefore advise caution in interpreting our results. Also, additional analyses on a subgroup of ever smokers could have provided valuable insights into the interaction between hormonal factors and smoking, particularly given that lung and UADT cancers are generally more frequent among ever smokers. Indeed, ever smokers stand to benefit substantially from targeted prevention strategies. However, in our cohort, heterogeneity in smoking behaviours (e.g., age at initiation, intensity, duration, cessation) and the relatively modest number of cases among ever smokers limited our ability to conduct adequately powered and interpretable stratified analyses. We acknowledge, however, that this strategy may limit the broader generalizability of our findings, and future studies should conduct analyses in subgroups using detailed smoking data to investigate these associations among smokers.

We acknowledge that UADT cancers are heterogeneous in terms of aetiology (e.g., tobacco, oral HPV); therefore, the risk from hormonal exposure may not be adequately represented by grouping all subsites. In our analysis, we grouped upper aerodigestive tract (UADT) cancers together, including non‐squamous cell carcinomas, HPV‐positive, salivary glands and oesophageal cancers, despite the distinct aetiologies of these subtypes. These subtypes may differ in their associations with hormonal factors, which may limit the interpretation of our findings. We combined these cancers to preserve statistical power and precision in our measures. This approach represents a necessary compromise since the incidence of lung and UADT cancers in women is low relative to the incidence in men and other cancer types (e.g., breast and colon). Precision is further hampered when we restrict the sample to never smokers. Consequently, this produces measures of association with marginal statistical significance that should be interpreted with caution.

Our study has some notable strengths. The E3N study has a robust prospective design and data collection tools; we had the advantage of substantially reducing recall bias. For our analyses, we introduced a missing category for certain variables in the regression models to ensure better comparisons between analyses. Since cancer cases were validated after a rigorous process, we minimised problems with case classification bias. The restriction of our analyses to never smokers ensured the control of the known confounding effect of tobacco smoking. This strategy was especially important and novel for UADT cancer aetiology since previous studies had small samples of incident cancers in women [5, 21, 27]; thus, analyses restricted to never smokers with reasonable statistical power have been uncommon [10]. Hence, with the addition of our study, we augment the small list of cohorts that have been able to study the risk of UADT cancers among females and among the first to achieve this among never smokers.

For future analyses, more parsimonious models should be built to estimate the effects of exposure factors to optimise the often‐limited statistical power usually observed in this area of study. In addition, the dynamic evolution in lifetime hormonal exposure should be taken into account using time‐dependent variables in regression models or similar methods. Furthermore, the types of exogenous hormonal treatments taken by women should be considered in future analyses. There are previous studies that showed a difference in lung cancer risk according to the type of treatment (e.g., contraceptive rings, implants, patches, IUDs) and not just limited to oral contraception [55]. Our primary focus was on hormonal factors linked to oestrogen, given its well‐established role in the development of various cancers, including breast and other reproductive cancers [56]. However, it is important to acknowledge that other hormones, such as testosterone and non‐sex hormones, may play a role in the incidence of lung and UADT cancers and potentially explain sex disparities. Considering the sample size limitations, we encourage the replication of this study in larger pooled analyses to confirm these findings.

## 5 Conclusion

Despite the limited precision in our measures, our French cohort study showed a few noteworthy associations. Shorter menstrual cycles (< 24 days) was associated with a higher risk of developing female lung cancer overall and among never smokers. Among never smokers, the risk of UADT cancer was 60% lower among women with at least 3 pregnancies compared to nulligravidae. The associations we observed for lung cancer do not align with the commonly adopted hypothesis of oestrogen deficiency as a mechanism for disease risk [9]. While larger studies are needed to elucidate the interactions between individual hormonal factors and known risk factors (tobacco, alcohol, BMI etc.), these findings are novel, particularly for UADT cancer since our study is one of the first longitudinal studies among female never smokers.

## Author Contributions

**Yawo E. Klu:** data curation (equal), formal analysis (equal), investigation (equal), methodology (equal), writing – original draft (equal), writing – review and editing (equal). **Hélène Amazouz:** formal analysis (supporting), investigation (equal), methodology (equal), supervision (supporting), writing – review and editing (lead). **Marianne Canonico:** methodology (supporting), writing – review and editing (equal). **Pascal Guénel:** methodology (equal), writing – review and editing (equal). **Marina Kvaskoff:** conceptualization (supporting), data curation (supporting), writing – review and editing (supporting). **Gianluca Severi:** resources (supporting), writing – review and editing (supporting). **Loredana Radoi:** methodology (equal), writing – review and editing (equal). **Aviane Auguste:** conceptualization (lead), data curation (lead), formal analysis (equal), funding acquisition (lead), investigation (lead), methodology (lead), project administration (lead), resources (lead), software (lead), supervision (lead), validation (lead), visualization (lead), writing – original draft (lead), writing – review and editing (lead).

## Conflicts of Interest

The authors declare no conflicts of interest.

## Supporting information


**Data S1.** Supporting Information.

## Data Availability

The datasets generated and/or analyzed during the current study are not publicly available since personal health data underlying the findings are protected by the French Data Protection Act. Data are however available from the corresponding author on reasonable request.
