# Supplementary material for: Association Between Hormonal Factors and Risk of Lung and Upper Aerodigestive Tract Cancer in French Women: The E3N Prospective Cohort Study
Source: Cancer Rep (Hoboken). 2025 Jun 9;8(6):e70223. doi: 10.1002/cnr2.70223 (PMC12148956; doi:10.1002/cnr2.70223)
Supplement: Supplementary file 1 — Data S1. Supporting Information. [file CNR2-8-e70223-s001.docx]

**Title:** Association between hormonal factors and risk of lung and upper aerodigestive tract cancer in French women: the E3N prospective cohort study

**Authors**

Yawo E. Klu, Hélène Amazouz, Marianne Canonico, Pascal Guénel, Marina Kvaskoff, Gianluca Severi, Loredana Radoi, Aviane Auguste

**Table S1:** Univariate relative risk (RR) and 95% CI of lung and UADT cancer from log-binomial model associated with hormonal factors among never smokers.

**Table S2**: Multivariate HR of lung and UADT cancer and 95% CI associated with sociodemographics and suspect/known risk factors (other covariates)

**Table S3:** Multivariate HR and 95% CI of lung and UADT cancer associated with hormonal factors (complete case analysis).

**Table S4:** Multivariate HR of respiratory cancers (lung and UADT combined) and 95% CI associated with hormonal factors.

**Table S5:** Summary of timing of individual variables in the current analysis

**Table S1:** Univariate relative risk (RR) and 95% CI of lung and UADT cancer from log-binomial model associated with hormonal factors among never smokers.

| Characteristic | Lung and UADT (*n* = 219) | | |  | Lung (*n* = 152) | | |  | UADT (*n* = 67) | | |
| --- | --- | --- | --- | --- | --- | --- | --- | --- | --- | --- | --- |
|  | *n* (row%) | RR | 95% CI |  | *n* (row%) | RR | 95% CI |  | *n* (row%) | RR | 95% CI |
| *Endogenous factors* |  |  |  |  |  |  |  |  |  |  |  |
| Age at menarche (y) |  |  |  |  |  |  |  |  |  |  |  |
| <12 | 46 (0.48) | 1.08 | 0.76-1.51 |  | 29 (0.30) | 0.89 | 0.57-1.34 |  | 17 (0.18) | 1.71 | 0.91-3.14 |
| 12-13 | 107 (0.45) | 1 | ref |  | 82 (0.34) | 1 | ref |  | 25 (0.10) | 1 | ref |
| ≥ 14 | 64 (0.46) | 1.03 | 0.75-1.40 |  | 40 (0.29) | 0.84 | 0.57-1.21 |  | 24 (0.17) | 1.65 | 0.94-2.89 |
| Menstrual cycle length |  |  |  |  |  |  |  |  |  |  |  |
| ≤24 days | 20 (0.72) | 1.65 | 1.00-2.56 |  | 15 (0.54) | 1.75 | 0.98-2.92 |  | 5 (0.18) | 1.39 | 0.48-3.20 |
| 25-31 days | 142 (0.44) | 1 | ref |  | 100 (0.31) | 1 | ref |  | 42 (0.13) | 1 | ref |
| ≥32 days | 18 (0.40) | 0.91 | 0.54-1.44 |  | 11 (0.24) | 0.79 | 0.40-1.40 |  | 7 (0.15) | 1.20 | 0.49-2.49 |
| Irregular | 31 (0.46) | 1.05 | 0.70-1.52 |  | 22 (0.32) | 1.05 | 0.65-1.64 |  | 9 (0.13) | 1.03 | 0.47-2.01 |
| Age at menopause (y) |  |  |  |  |  |  |  |  |  |  |  |
| <40 | 3 (0.30) | 0.70 | 0.17-1.83 |  | 3 (0.30) | 1.01 | 0.25-2.67 |  | 0 (0.00) |  |  |
| 41-45 | 18 (0.65) | 1.50 | 0.89-2.36 |  | 12 (0.43) | 1.44 | 0.76-2.50 |  | 6 (0.22) | 1.62 | 0.70-3.77 |
| 46-55 | 172 (0.43) | 1 | ref |  | 119 (0.30) | 1 | ref |  | 53 (0.13) | 1 | ref |
| ≥55 | 20 (0.50) | 1.15 | 0.70-1.78 |  | 13 (0.32) | 1.08 | 0.58-1.84 |  | 7 (0.17) | 1.30 | 0.59-2.87 |
| Duration of reproductive life (y) |  |  |  |  |  |  |  |  |  |  |  |
| <32 | 14 (0.47) | 0.96 | 0.49-1.79 |  | 10 (0.34) | 0.87 | 0.40-1.79 |  | 4 (0.13) | 1.28 | 0.33-4.48 |
| 32-35 | 41 (0.48) | 0.99 | 0.61-1.61 |  | 25 (0.29) | 0.77 | 0.43-1.37 |  | 16 (0.19) | 1.80 | 0.74-5.00 |
| 36-38 | 62 (0.39) | 0.80 | 0.52-1.27 |  | 44 (0.28) | 0.72 | 0.44-1.23 |  | 18 (0.11) | 1.09 | 0.46-2.99 |
| 39-41 | 66 (0.48) | 0.99 | 0.64-1.56 |  | 45 (0.33) | 0.86 | 0.52-1.45 |  | 21 (0.15) | 1.47 | 0.63-4.00 |
| ≥ 42 | 28 (0.49) | 1 | ref |  | 22 (0.38) | 1 | ref |  | 6 (0.10) | 1 | ref |
| Number of children |  |  |  |  |  |  |  |  |  |  |  |
| 0 | 27 (0.51) | 1 | ref |  | 17 (0.32) | 1 | ref |  | 10 (0.19) | 1 | ref |
| 1 | 30 (0.41) | 0.80 | 0.47-1.35 |  | 19 (0.26) | 0.80 | 0.42-1.56 |  | 11 (0.15) | 0.79 | 0.33-1.89 |
| 2 | 95 (0.46) | 0.90 | 0.60-1.41 |  | 67 (0.33) | 1.01 | 0.61-1.77 |  | 28 (0.14) | 0.72 | 0.36-1.55 |
| ≥3 | 67 (0.43) | 0.85 | 0.55-1.34 |  | 49 (0.32) | 0.98 | 0.58-1.75 |  | 18 (0.12) | 0.61 | 0.29-1.38 |
| Number of pregnancies |  |  |  |  |  |  |  |  |  |  |  |
| 0 | 23 (0.53) | 1 | ref |  | 13 (0.30) | 1 | ref |  | 10 (0.23) | 1 | ref |
| 1 | 25 (0.42) | 0.80 | 0.45-1.41 |  | 16 (0.27) | 0.90 | 0.44-1.91 |  | 9 (0.15) | 0.66 | 0.26-1.64 |
| 2 | 76 (0.48) | 0.92 | 0.59-1.50 |  | 54 (0.34) | 1.15 | 0.65-2.21 |  | 22 (0.14) | 0.61 | 0.30-1.35 |
| ≥3 | 95 (0.42) | 0.79 | 0.51-1.28 |  | 69 (0.30) | 1.02 | 0.58-1.93 |  | 26 (0.11) | 0.50 | 0.25-1.09 |
| *Exogenous factors* |  |  |  |  |  |  |  |  |  |  |  |
| Oral contraception |  |  |  |  |  |  |  |  |  |  |  |
| Never | 111 (0.49) | 1 | ref |  | 74 (0.33) | 1 | ref |  | 37 (0.16) | 1 | ref |
| Ever | 108 (0.42) | 0.85 | 0.65-1.11 |  | 78 (0.30) | 0.92 | 0.67-1.27 |  | 30 (0.12) | 0.71 | 0.44-1.15 |
| HRT |  |  |  |  |  |  |  |  |  |  |  |
| Never | 82 (0.45) | 1 | ref |  | 53 (0.29) | 1 | ref |  | 29 (0.16) | 1 | ref |
| Ever | 137 (0.45) | 0.99 | 0.76-1.31 |  | 99 (0.32) | 1.11 | 0.80-1.56 |  | 38 (0.12) | 0.78 | 0.48-1.27 |

HRT: Hormone replacement therapy

Analyses performed using missing category for certain variables (*n*=91,114 participants).

E3N cohort study, France 1990-2014.

**Table S2**: Multivariate HR of lung and UADT cancer and 95% CI associated with sociodemographics and suspect/known risk factors (other covariates)

|  | Overall | | | | | | |  | Never smokers | | | | | | |
| --- | --- | --- | --- | --- | --- | --- | --- | --- | --- | --- | --- | --- | --- | --- | --- |
| Characteristic | Lung (*n* =398) | | |  | UADT (*n* =157) | | |  | Lung (*n* =152) | | |  | UADT (*n* =67) | | |
|  | *n* (row%) | HR | 95% CI |  | *n* (row%) | HR | 95% CI |  | *n* (row%) | HR | 95% CI |  | *n* (row%) | HR | 95% CI |
| **Age at inclusion** |  |  |  |  |  |  |  |  |  |  |  |  |  |  |  |
| <49 | 123 (0.28) | ref |  |  | 60 (0.14) | ref |  |  | 37 (0.18) | ref |  |  | 20 (0.10) | ref |  |
| ≥ 49 | 275 (0.57) | 1.2 | (0.92-1.56) |  | 97 (0.20) | 0.87 | (0.57-1.33) |  | 115 (0.41) | 1.29 | (0.82-2.04) |  | 47 (0.17) | 0.81 | (0.42-1.57) |
| **Town of work** |  |  |  |  |  |  |  |  |  |  |  |  |  |  |  |
| Rural | 170 (0.47) | ref |  |  | 79 (0.22) | ref |  |  | 77 (0.37) | ref |  |  | 40 (0.19) | ref |  |
| Urban | 119 (0.40) | 1.07 | (0.84-1.37) |  | 42 (0.14) | 0.67 | (0.45-1.01) |  | 39 (0.27) | 0.99 | (0.66-1.49) |  | 15 (0.10) | 0.66 | (0.35-1.23) |
| **Education level** |  |  |  |  |  |  |  |  |  |  |  |  |  |  |  |
| Primary | 17 (0.39) | 0.92 | (0.56-1.52) |  | 5 (0.11) | 0.71 | (0.28-1.76) |  | 9 (0.28) | 0.90 | (0.45-1.8) |  | 4 (0.13) | 0.94 | (0.33-2.66) |
| Secondary | 31 (0.40) | 0.92 | (0.64-1.34) |  | 22 (0.28) | 1.63 | (1.01-2.63) |  | 13 (0.27) | 0.85 | (0.48-1.51) |  | 10 (0.21) | 1.62 | (0.81-3.22) |
| Tertiary | 339 (0.45) | ref |  |  | 127 (0.17) | ref |  |  | 123 (0.32) | ref |  |  | 52 (0.13) | ref |  |
| **Western diet** |  |  |  |  |  |  |  |  |  |  |  |  |  |  |  |
| Q1-Q2 | 151 (0.43) | ref |  |  | 67 (0.19) | ref |  |  | 68 (0.34) | ref |  |  | 31 (0.16) | ref |  |
| Q3-Q4 | 161 (0.45) | 1.18 | (0.94-1.5) |  | 60 (0.17) | 0.87 | (0.6-1.26) |  | 54 (0.30) | 0.99 | (0.68-1.45) |  | 28 (0.15) | 0.98 | (0.57-1.68) |
| **Mediterranean diet** |  |  |  |  |  |  |  |  |  |  |  |  |  |  |  |
| Q1-Q2 | 163 (0.46) | ref |  |  | 54 (0.15) | ref |  |  | 66 (0.34) | ref |  |  | 25 (0.13) | ref |  |
| Q3-Q4 | 149 (0.42) | 0.86 | (0.69-1.07) |  | 73 (0.21) | 1.32 | (0.92-1.89) |  | 56 (0.30) | 0.84 | (0.58-1.2) |  | 34 (0.18) | 1.44 | (0.85-2.44) |
| **Tobacco smoking** |  |  |  |  |  |  |  |  |  |  |  |  |  |  |  |
| Never | 246 (0.58) | ref |  |  | 90 (0.21) | ref |  |  | NA | NA | NA |  | NA | NA | NA |
| Ever | 152 (0.31) | **2.01** | (1.63-2.48) |  | 67 (0.14) | 1.55 | (1.11-2.16) |  | NA | NA | NA |  | NA | NA | NA |
| **Alcohol drinking (g/day)** |  |  |  |  |  |  |  |  |  |  |  |  |  |  |  |
| <5 | 119 (0.39) | 0.97 | (0.75-1.24) |  | 40 (0.13) | 0.55 | (0.37-0.83) |  | 54 (0.28) | 0.77 | (0.53-1.13) |  | 20 (0.11) | 0.52 | (0.29-0.92) |
| 5 -24 | 139 (0.45) | ref |  |  | 69 (0.22) | ref |  |  | 58 (0.38) | ref |  |  | 32 (0.21) | ref |  |
| ≥25 | 54 (0.56) | 1.11 | (0.8-1.53) |  | 18 (0.19) | 0.78 | (0.45-1.33) |  | 10 (0.28) | 0.76 | (0.39-1.49) |  | 7 (0.19) | 0.99 | (0.43-2.26) |
| **BMI** |  |  |  |  |  |  |  |  |  |  |  |  |  |  |  |
| <25 | 62 (0.39) | ref |  |  | 30 (0.19) | ref |  |  | 25 (0.29) | ref |  |  | 15 (0.17) | ref |  |
| ≥25 | 325 (0.44) | 1.23 | (0.93-1.63) |  | 123 (0.17) | 0.92 | (0.6-1.39) |  | 121 (0.31) | 1.11 | (0.71-1.73) |  | 51 (0.13) | 0.87 | (0.47-1.61) |
| **Physical activity** |  |  |  |  |  |  |  |  |  |  |  |  |  |  |  |
| Inactive | 12 (0.59) | 1.85 | (0.93-3.69) |  | 4 (0.20) | 1.24 | (0.4-3.86) |  | 5 (0.44) | 2.95 | (0.9-9.7) |  | 0 (0.00) | NA | NA |
| Moderate | 25 (0.33) | ref |  |  | 12 (0.16) | ref |  |  | 6 (0.15) | ref |  |  | 3 (0.08) | ref |  |
| Vigorous | 352 (0.44) | 1.29 | (0.86-1.93) |  | 140 (0.17) | 1.00 | (0.55-1.8) |  | 135 (0.31) | 1.95 | (0.86-4.43) |  | 64 (0.15) | 1.8 | (0.56-5.77) |

Model adjusted for all variables listed in the table plus age at menarche, number of pregnancies, age at menopause, menstrual cycle length, hormone replacement therapy and oral contraceptive.

HRT: Hormone replacement therapy

Analyses performed using missing category for certain variables (*n*=91,075 participants).

E3N cohort study, France 1990-2014.

**Table S3:** Multivariate HR and 95% CI of lung and UADT cancer associated with hormonal factors (complete case analysis).

| Hormonal factor | Overall | | |  | Never smokers | | |
| --- | --- | --- | --- | --- | --- | --- | --- |
|  | Lung (*n* = 243) |  | UADT (*n* = 100) |  | Lung (*n* = 97) |  | UADT (*n* = 47) |
|  | HR (95% CI) |  | HR (95% CI) |  | HR (95% CI) |  | HR (95% CI) |
| *Endogenous factors* |  |  |  |  |  |  |  |
| **Age at menarche (y)** |  |  |  |  |  |  |  |
| <12 | 0.84 (0.60-1.17) |  | 0.96 (0.55-1.68) |  | 0.77 (0.45-1.33) |  | 1.88 (0.93-3.82) |
| 12-13 | ref |  | ref |  | ref |  | ref |
| ≥ 14 | 0.68 (0.50-0.93) |  | 1.51 (0.97-2.33) |  | 0.64 (0.39-1.04) |  | 1.26 (0.64-2.5) |
| **Number of pregnancies** |  |  |  |  |  |  |  |
| 0 | ref |  | ref |  | ref |  | ref |
| 1 | 1.22 (0.68-2.17) |  | 0.98 (0.44-2.17) |  | 0.87 (0.37-2.02) |  | 1.06 (0.33-3.38) |
| 2 | 1.27 (0.76-2.11) |  | 0.56 (0.27-1.18) |  | 0.93 (0.46-1.91) |  | 0.81 (0.29-2.26) |
| ≥3 | 1.19 (0.73-1.95) |  | 0.92 (0.47-1.79) |  | 0.66 (0.32-1.35) |  | 0.67 (0.24-1.82) |
| **Age at menopause (y)** |  |  |  |  |  |  |  |
| <40 | 1.36 (0.64-2.91) |  | NA |  | 1.53 (0.48-4.9) |  | NA |
| 41-45 | 1.64 (1.06-2.53) |  | 1.57 (0.79-3.15) |  | 1.26 (0.58-2.73) |  | 1.49 (0.53-4.2) |
| 46-55 | ref |  | ref |  | ref |  | ref |
| ≥55 | 0.79 (0.47-1.35) |  | 1.11 (0.56-2.24) |  | 1.3 (0.65-2.6) |  | 1.69 (0.71-4.06) |
| **Menstrual cycle length** |  |  |  |  |  |  |  |
| ≤24 days | 1.44 (0.90-2.29) |  | 0.98 (0.43-2.27) |  | 1.83 (0.94-3.57) |  | 0.66 (0.16-2.75) |
| 25-31 days | ref |  | ref |  | ref |  | ref |
| ≥32 days | 1.37 (0.91-2.06) |  | 1.12 (0.59-2.13) |  | 1.32 (0.67-2.58) |  | 1.5 (0.62-3.61) |
| Irregular | 1.25 (0.87-1.78) |  | 0.75 (0.40-1.43) |  | 1.32 (0.75-2.33) |  | 0.79 (0.31-2.04) |
| *Exogenous factors* |  |  |  |  |  |  |  |
| **HRT** |  |  |  |  |  |  |  |
| Never | ref |  | ref |  | ref |  | ref |
| Ever | 1.13 (0.84-1.52) |  | 1.01 (0.65-1.56) |  | 1.54 (0.95-2.5) |  | 0.86 (0.46-1.6) |
| **Oral contraceptive** |  |  |  |  |  |  |  |
| Never | ref |  | ref |  | ref |  | ref |
| Ever | 1.26 (0.95-1.68) |  | 0.98 (0.63-1.53) |  | 1.51 (0.97-2.34) |  | 1.17 (0.62-2.22) |
| *Other covariates* |  |  |  |  |  |  |  |
| **Age at inclusion** |  |  |  |  |  |  |  |
| <49 | ref |  |  |  | ref |  | ref |
| ≥ 49 | 0.99 (0.70-1.39) |  | 0.89 (0.52-1.51) |  | 1.33 (0.74-2.37) |  | 1.05 (0.47-2.38) |
| **Town of work** |  |  |  |  |  |  |  |
| Rural | ref |  | ref |  | ref |  | ref |
| Urban | 0.94 (0.72-1.23) |  | 0.78 (0.50-1.21) |  | 0.87 (0.55-1.36) |  | 0.71 (0.36-1.41) |
| **Education level** |  |  |  |  |  |  |  |
| Primary | 0.8 (0.37-1.71) |  | 0.8 (0.25-2.58) |  | 0.57 (0.18-1.83) |  | 1.23 (0.37-4.07) |
| Secondary | 0.76 (0.45-1.28) |  | 1.54 (0.84-2.84) |  | 0.73 (0.34-1.58) |  | 1.43 (0.60-3.42) |
| Tertiary | ref |  | ref |  | ref |  | ref |
| **Western diet** |  |  |  |  |  |  |  |
| Q1-Q2 | ref |  | ref |  | ref |  | ref |
| Q3-Q4 | 1.1 (0.84-1.44) |  | 0.86 (0.57-1.31) |  | 1.01 (0.66-1.55) |  | 1.01 (0.55-1.85) |
| **Mediterranean diet** |  |  |  |  |  |  |  |
| Q1-Q2 | ref |  | ref |  | ref |  | ref |
| Q3-Q4 | 0.88 (0.69-1.13) |  | 1.2 (0.81-1.78) |  | 0.92 (0.62-1.37) |  | 1.26 (0.7-2.24) |
| **Tobacco smoking** |  |  |  |  |  |  |  |
| Never | ref |  | ref |  | NA |  | NA |
| Ever | 1.88 (1.45-2.45) |  | 1.39 (0.93-2.09) |  | NA |  | NA |
| **Alcohol drinking** |  |  |  |  |  |  |  |
| <5 | 1.02 (0.77-1.36) |  | 0.57 (0.36-0.89) |  | 0.92 (0.6-1.4) |  | 0.55 (0.29-1.05) |
| 5 -24 | ref |  | ref |  | ref |  | ref |
| ≥25 | 1.25 (0.87-1.78) |  | 0.82 (0.45-1.49) |  | 0.93 (0.45-1.93) |  | 1.31 (0.56-3.07) |
| **BMI** |  |  |  |  |  |  |  |
| ≥25 | ref |  | ref |  | 1.42 (0.77-2.64) |  | 1.18 (0.54-2.59) |
| <25 | 1.25 (0.87-1.81) |  | 1.01 (0.59-1.73) |  | ref |  | ref |
| **Physical activity** |  |  |  |  |  |  |  |
| Inaccurate | 1.2 (0.44-3.27) |  | 0.52 (0.06-4.2) |  | 1.86 (0.34-10.18) |  | NA |
| Moderate | ref |  | ref |  | ref |  | Ref |
| Vigorous | 1.18 (0.71;1.95) |  | 1.04 (0.48;2.25) |  | 1.81 (0.66;4.93) |  | 1.67 (0.4;6.9) |

Model adjusted for all variables listed in the table

Complete case analysis: Analyses performed without introducing missing categories for certain covariables (*n*= 56,303).

E3N cohort study, France 1990-2014.

**Table S4:** Multivariate HR of respiratory cancers (lung and UADT combined) and 95% CI associated with hormonal factors.

|  | Lung and UADT cancer | | |
| --- | --- | --- | --- |
|  | Overall |  | Never smokers |
|  | HR (95% CI) |  | HR (95% CI) |
| *Endogenous factors* |  |  |  |
| **Age at menarche (y)** |  |  |  |
| <12 | 0.89 (0.71-1.11) |  | 1.12 (0.79-1.58) |
| 12-13 | ref |  | ref |
| ≥ 14 | 0.92 (0.75-1.12) |  | 0.98 (0.72-1.34) |
| **Number of pregnancies** |  |  |  |
| 0 | ref |  | ref |
| 1 | 1.14 (0.8-1.64) |  | 0.84 (0.47-1.49) |
| 2 | 0.97 (0.7-1.35) |  | 0.95 (0.59-1.54) |
| ≥3 | 1.02 (0.75-1.4) |  | 0.74 (0.46-1.18) |
| **Age at menopause (y)** |  |  |  |
| <40 | 0.77 (0.40-1.50) |  | 0.62 (0.2-1.96) |
| 41-45 | 1.82 (1.38-2.41) |  | 1.4 (0.86-2.29) |
| 46-55 | 0.72 (0.50-1.05) |  | ref |
| ≥55 | ref |  | 1.18 (0.73-1.9) |
| **Menstrual cycle length** |  |  |  |
| ≤24 days | 1.44 (1.05-1.97) |  | 1.64 (1.03-2.63) |
| 25-31 days | ref |  | ref |
| ≥32 days | 1.03 (0.75-1.4) |  | 1.04 (0.64-1.71) |
| Irregular | 1.19 (0.94-1.5) |  | 1.10 (0.74-1.63) |
| *Exogenous factors* |  |  |  |
| **HRT** |  |  |  |
| Never | ref |  | ref |
| Ever | 0.91 (0.76-1.1) |  | 1.01 (0.75-1.36) |
| **Oral contraceptive** |  |  |  |
| Never | ref |  | ref |
| Ever | 1.19 (0.98-1.45) |  | 1.25 (0.93-1.68) |
| *Other covariates* |  |  |  |
| **Age at inclusion** |  |  |  |
| <49 | ref |  | ref |
| ≥ 49 | 1.10 (0.88-1.38) |  | 1.11 (0.77-1.62) |
| **Town of work** |  |  |  |
| Rural | ref |  | ref |
| Urban | 0.94 (0.76-1.16) |  | 0.87 (0.62-1.23) |
| **Education level** |  |  |  |
| Primary | 0.86 (0.56-1.34) |  | 0.90 (0.51-1.61) |
| Secondary | 1.11 (0.83-1.49) |  | 1.07 (0.69-1.66) |
| Tertiary | ref |  | ref |
| **Western diet** |  |  |  |
| Q1-Q2 | ref |  | ref |
| Q3-Q4 | 1.09 (0.89-1.33) |  | 0.99 (0.73-1.35) |
| **Mediterranean diet** |  |  |  |
| Q1-Q2 | ref |  | ref |
| Q3-Q4 | 0.97 (0.8-1.17) |  | 1.00 (0.74-1.34) |
| **Tobacco smoking** |  |  |  |
| Never | ref |  | NA |
| Ever | 1.86 (1.56-2.22) |  | NA |
| **Alcohol drinking (g/day)** |  |  |  |
| <5 | 0.83 (0.67-1.02) |  | 0.68 (0.5-0.94) |
| 5 -24 | ref |  | ref |
| ≥25 | 1.00 (0.76-1.32) |  | 0.84 (0.5-1.42) |
| **BMI** |  |  |  |
| ≥25 | 1.13 (0.9-1.42) |  | 1.03 (0.72-1.47) |
| <25 | ref |  | ref |
| **Physical activity** |  |  |  |
| Inaccurate | 1.65 (0.92-2.98) |  | 1.92 (0.64-5.73) |
| Moderate | ref |  | ref |
| Vigorous | 1.19 (0.85-1.67) |  | 1.91 (0.98-3.73) |

Model adjusted for all variables listed in the table

Analyses performed using missing category for certain variables (*n*=91,075 participants).

E3N cohort study, France 1990-2014.

**Table S5:** Summary of timing of individual variables in the current analysis

| Variables in the study | Q1 | Q3 | Q11 |
| --- | --- | --- | --- |
| **Outcome variable** |  |  |  |
| Cancer incidence |  |  | X |
| **Covariates** |  |  |  |
| Age at inclusion | X |  |  |
| Town of work | X |  |  |
| Education level |  |  |  |
| Western diet |  | X |  |
| Mediterranean diet |  | X |  |
| Tobacco smoking | X |  |  |
| Alcohol drinking (g/day) |  | X |  |
| BMI | X |  |  |
| Physical activity | X |  |  |
| **Hormonal factors** |  |  |  |
| Age at menarche (y) | X |  |  |
| Menstrual cycle length (days) | X |  |  |
| Age at menopause (y) | X |  |  |
| Duration of reproductive life (y) | X |  |  |
| Number of children | X |  |  |
| Number of pregnancies | X |  |  |
| Oral contraceptive | X |  |  |
| Hormone replacement therapy | X |  |  |
